# Electrical, Structural, Optical, and Adhesive Characteristics of Aluminum-Doped Tin Oxide Thin Films for Transparent Flexible Thin-Film Transistor Applications

**DOI:** 10.3390/ma12010137

**Published:** 2019-01-03

**Authors:** Seung-Hun Lee, Kihwan Kwon, Kwanoh Kim, Jae Sung Yoon, Doo-Sun Choi, Yeongeun Yoo, Chunjoong Kim, Shinill Kang, Jeong Hwan Kim

**Affiliations:** 1 Department of Nano Manufacturing Technology, Korea Institute of Machinery and Materials (KIMM), Daejeon 34103, Korea; qwpo45ei@kimm.re.kr (S.-H.L.); nankkh@kimm.re.kr (K.K.); kkim@kimm.re.kr (K.K.); jaesyoon@kimm.re.kr (J.S.Y.); choids@kimm.re.kr (D.-S.C.); yeyoo@kimm.re.kr (Y.Y.); 2 Department of Materials Science and Engineering, Chungnam National University, Daejeon 34134, Korea; ckim0218@cnu.ac.kr; 3 School of Mechanical Engineering, Yonsei University, Seoul 03722, Korea; 4 Department of Nano-Mechatronics, University of Science and Technology, Daejeon 34113, Korea

**Keywords:** thin-film transistor, tin oxide, aluminum doping, oxide semiconductor, adhesive property

## Abstract

The properties of Al-doped SnO_x_ films deposited via reactive co-sputtering were examined in terms of their potential applications for the fabrication of transparent and flexible electronic devices. Al 2.2-atom %-doped SnO_x_ thin-film transistors (TFTs) exhibit improved semiconductor characteristics compared to non-doped films, with a lower sub-threshold swing of ~0.68 Vdec^−1^, increased on/off current ratio of ~8 × 10^7^, threshold voltage (V_th_) near 0 V, and markedly reduced (by 81%) V_th_ instability in air, attributable to the decrease in oxygen vacancy defects induced by the strong oxidizing potential of Al. Al-doped SnO_x_ films maintain amorphous crystallinity, an optical transmittance of ~97%, and an adhesive strength (to a plastic substrate) of over 0.7 kgf/mm; such films are thus promising semiconductor candidates for fabrication of transparent flexible TFTs.

## 1. Introduction

In recent years, intensive research efforts have focused on metal oxide semiconductors, such as ZnO, SnO_2_, In_2_O_5_, and InGaZnO_5_, which have many applications in electronics, sensors, and active matrix displays. Because of their high electron mobility and good transparency, metal oxide semiconductors are especially ideal candidates for the active layers of thin-film transistors (TFTs) [1,2,3,4,5,6,7]. Most metal oxide semiconductors are fabricated via sputtering; their properties are determined by reactions between the injected gases (Ar and O_2_) and the metals (e.g., In, Sn, Zn, and Ga), typically yielding amorphous, conductive, and transparent materials. Especially compared to amorphous silicon, metal oxide semiconductors can be fabricated at a relatively low temperature (≤300 °C), even room temperature, and have higher electron mobility than amorphous silicon, thus finding many applications in the manufacture of organic light-emitting diode display panels.

However, metal oxide semiconductors have a problem of stability, because of oxygen deficiencies and reaction with the external environment. The high electron carrier level caused by oxygen vacancies also compromises semiconductor device controllability. Therefore, it is very important to reduce oxygen vacancy defects. If a doped component could maintain the oxygen content in the metal oxide semiconductors, the device stability and controllability would improve [8].

Here, we fabricated Al-doped SnO_x_ films via sputtering and explored the effect of the Al doping on SnOx films on electrical, structural, and optical properties. We further fabricated TFTs to investigate their transfer characteristics and stability. In addition, we used a simple peel test to estimate adhesion between Al-doped SnO_x_ thin films and flexible plastic substrates at different Al contents, which would be critical to flexible device fabrication [9,10,11].

## 2. Materials and Methods

Bottom gate TFTs based on Al-doped SnO_x_ were fabricated. Highly doped Si (P^++^) and thermally grown SiO_2_ (100 nm) served as the gate electrode and insulator, respectively. As a channel layer, 10 nm thick SnO_2_ films were deposited using radiofrequency (RF) reactive magnetron sputtering. For Al doping of SnO_2_, co-sputtering was performed using Sn (99.999%) and Al (99.999%) targets and an O_2_/Ar gas mixture (O_2_:Ar = 1:3). To explore the effects of doping, the input RF power of the Al target was varied from 0 to 100 and 200 W (yielding TFTs termed SO, ASO1, and ASO2, respectively), whereas that of the Sn target was fixed at 200 W. The channel thickness of all three samples was fixed at 10 nm, as confirmed using a spectroscopic ellipsometer (MG-1000, Nano-View Co., Ansan, Korea) and a field effect scanning electron microscope (S5300, Hitachi Inc., Tokyo, Japan). The source/drain electrodes were 10 wt % Sn-doped In_2_O_3_ (indium tin oxide), deposited via direct-current magnetron sputtering at 200 W to a thickness of 100 nm. 1000 μm wide and 100 μm long channel patterns were created using a shadow mask. Sputtering was performed at a base pressure of 1 × 10^−6^ Torr and a working pressure of 10 mTorr at room temperature. The TFTs were subjected to thermal annealing at 150 °C for 1 h in air using a quartz tube furnace. The electrical performance of the Al-doped SnO_x_ TFTs was measured using an HP4145B semiconductor analyzer operating at room temperature in the dark. In addition, we measured changes in TFT transfer characteristics over time. TFTs without passivation layers were exposed to the air at room temperature under dehumidifying condition. The chemical compositions and Sn-bonding states of the Al-doped SnO_x_ thin films were analyzed by X-ray photoelectron spectroscopy (XPS) (Multilab 2000, Thermo Scientific Ltd., Waltham, MA, USA). The atomic percentage of each element in the films was calculated by dividing the area under the curve by the relative sensitivity factor for each peak at C 1s, Al 2p, Sn 3d, and O 1s XPS spectra and normalizing the values over the total amount of the elements in the films. Carrier densities were measured using the Hall method (HMS-5300, Ecopia, Anyang, Korea), employing the Van der Pauw configuration. Crystallinity was evaluated via high-resolution X-ray diffraction (HR-XRD), during which Cu Kα radiation was delivered at a glancing angle (D8 Discover, Bruker AXS Ltd., Karlsruhe, Germany). Optical transmittance was assessed with the aid of a UV-visible spectrophotometer (UV-2450, Shimadzu Corp., Tokyo, Japan). A peel tester stand (AD4935-50N, And Inc., Tokyo, Japan) and duct tape (GT2, 3M Espe, Maplewood, NJ, USA) were used to measure adhesion between the Al-doped SnO_x_ thin films and the polyimide (PI) films. Samples were fixed to glass to prevent cracking during bending or handling. Changes in sheet resistance after the peel test were analyzed via four-point probing using a source meter (2400, Keithley Inc., Beaverton, AL, USA) to evaluate adhesion between the Al-doped SnO_x_ thin films and the PI films.

## 3. Results and Discussions

### 3.1. Electrical Characterization of Al-Doped SnO_x_ Thin-Film Transistors

**Figure 1:**
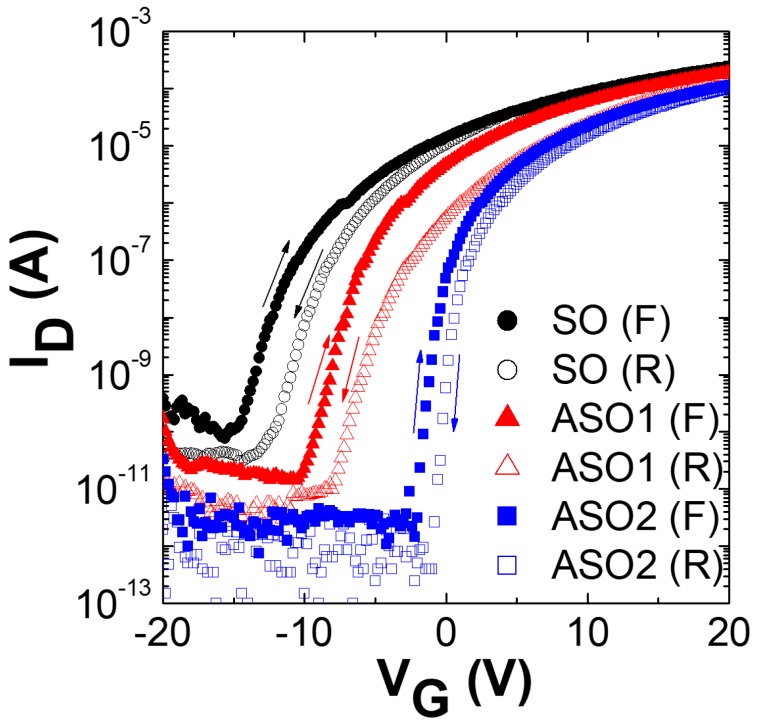
Transfer curves of the SO, ASO1 and ASO2 thin-film transistors (TFTs) containing Al at atom % values of 0, 1.20, and 2.24, which were scanned with forward (F) and reverse (R) bias sweeps (V_D_ = 10.1 V).

Figure 1 shows the transfer characteristics of SO, ASO1, and ASO2, which were scanned with forward (−20 V → +20 V) and reverse (+20 V → −20 V) bias sweeps. The source and drain voltages (V_D_) were the constant ground voltage and 10.1 V, respectively. The threshold voltage (V_th_) was the voltage evident when the current approached L/W × 10 nA [12]. The saturation mobility (μ_sat_) and subthreshold swing (SS) were 2L/WC_i_ × (dI_D_^1/2^/dV_G_)^2^ and (dlog I_D_/dV_G_)^−1^ respectively, where C_i_ is the capacitance per unit area of the gate dielectric (34.5 nF/cm^2^) [13,14,15]. The V_th_, I_on_/I_off_, μ_sat_, SS, and Al atomic concentrations (in atom %) of SO, ASO1, and ASO2 are listed in Table 1. The Al atomic percentage values were evaluated via XPS spectral analysis, assuming that the films consisted of C, Al, Sn, and O. As shown in Figure 1, the TFT device exhibited n-type conduction. Although SnO_2_ is a well-known n-type material, several papers have reported that Al-incorporated SnO_x_ showed p-type semiconducting behavior. This is due to the hole carriers produced by the Al^3+^-Sn^4+^ substitution reaction when the Al concentration exceeds 5% in a SnO_2_ film [16,17]. Therefore, it is possible the n-type behavior of the TFT may be caused by the low concentration (≤2.24%) of Al, which is not enough to make holes majority carriers in SnO_x_ films. As the extent of Al doping increased, V_th_ changed from −13.4 to −1.0 V, thus becoming close to 0 V, and the I_on_/I_off_ ratio increased dramatically (over 25-fold) because the off-current was much lower than that of undoped SnO_x_. Chu et al. reported that reduced carrier concentration in the n-type channel material induces higher V_th_ and lower I_off_, because the channel with lower carrier concentration is more easily depleted [18]. Therefore, these positive shifts of V_th_ and decreased I_off_ are attributable to reductions in electron density in the TFT channels as Al levels increase [2,6,18,19]. In general, oxygen vacancies in SnO_x_-based materials generate free electrons, which is accompanied by n-type semiconduction [20,21]. The standard oxidization potential (at 25 °C) of Al is higher than that of Sn by 1.37 V; Al is thus a stronger oxidizer than Sn [22]. Hence, if enough oxygen is supplied during the sputtering process, fewer oxygen vacancies are induced in Al-doped SnO_x_ layers compared to non-doped SnO_x_, because the film oxygen content is higher given the stronger oxidizing power of Al. Consequently, decreasing electron density with increasing Al content in the film could be attributed to fewer oxygen vacancies in the ASO1 and ASO2 channels due to the strong oxidizing potential of Al, resulting in the increased V_th_ and I_on_/I_off_ ratio. Moreover, the insulating property of Al_2_O_3_, which was formed during the RF sputtering process in the Ar/O_2_ ambience, could be another reason for lower electron density compared to non-doped SnO_x_ layer. Hall measurements confirmed the reductions in electron density, which were 9.41 × 10^18^ cm^−3^ for SO and 6.09 × 10^15^ cm^−3^ for ASO2. The μ_sat_ (2.27 ± 0.03 cm^2^ V^−1^ s^−1^) did not change significantly after doping, which was also confirmed by the similar slope in Figure S1 in the supplementary material. However, the SS of ASO2 fell to 0.68 V dec.^−1^, less than 33% of that of SO, further improving TFT performance. Clockwise hysteresis was observed, and revealed that electron traps existed at or near the channel/insulator interface. The hysteresis (|ΔV_th_|) of ASO2 was decreased to 1.13 V, much less than that of SO (2.67 V), which indicates that the hysteresis can be obviously suppressed by Al doping.

**Figure 2:**
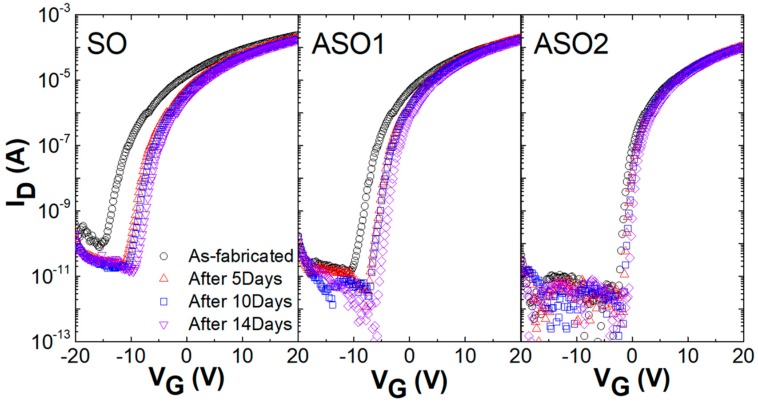
Changes in the transfer characteristics of SO, ASO1, and ASO2 TFTs (lacking passivation layers) over time.

We explored changes in TFT transfer characteristics over time (Figure 2). V_G_-I_D_ curves were drawn daily for 2 weeks after TFT fabrication; the V_th_ values gradually became more positive because the channels absorbed atmospheric oxygen [23,24]. After 14 days, the V_th_ values of SO, ASO1, and ASO2 had shifted by 6.2, 4.2, and 1.2 V, respectively, indicating that the ambient stability of SnO_x_ film can be improved by Al doping. Yan et al. reported that oxygen adsorption is preferred on oxygen-deficient surfaces, and that molecular oxygen can heal oxygen vacancies in the oxide material [25]. Therefore, this decreased V_th_ shift with increasing Al content is attributable to the reduced reaction between the surfaces of the channels and the oxygen in the atmosphere, because Al-doped channels have fewer oxygen vacancies than undoped channels, as discussed in Figure 1.

### 3.2. Structural and Optical Properties of Al-Doped SnO_x_ Thin-Films

**Figure 3:**
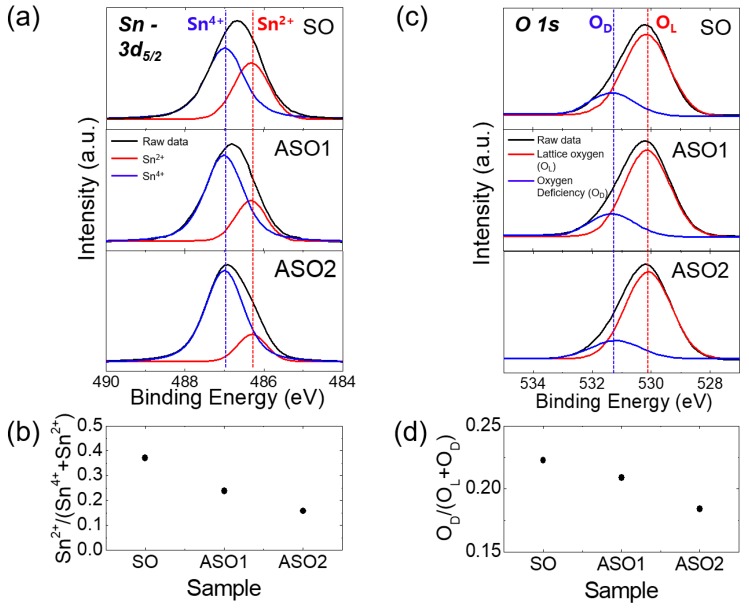
(**a**) Sn 3d_5/2_ X-ray photoemission spectroscopy (XPS) spectra of SO, ASO1, and ASO2 thin-films. (**b**) Relative peak intensities [Sn^2+^/Sn^4+^ + Sn^2+^] of the Sn^2+^ peaks. (**c**) O 1s XPS spectra of SO, ASO1, and ASO2 thin films. (**d**) Relative peak intensities of [O_D_/(O_L_ + O_D_)] of the O_D_ peaks.

In order to support the electrical characteristics of the TFTs, we performed analyses of structural and optical properties of SnO_x_ thin films with different Al concentrations. Figure 3a shows the Sn 3d_5/2_ XPS spectra of the SO, SAO1, and SAO2 films. The Sn 3d_5/2_ peaks were deconvoluted into two sub-peaks with binding energies of 487.0 and 486.3 eV [19,26], respectively, corresponding to the Sn^4+^ and Sn^2+^ binding states. The Sn^2+^ peak area clearly decreased with the Al proportion, while that of the Sn^4+^ peak increased. The relative peak intensity [Sn^2+^/Sn^4+^ + Sn^2+^] of the Sn^2+^ peak fell from 0.37 to 0.16 with increasing Al doping (Figure 3b). Huh et al. found that Sn^2+^ binding in SnO_2_ caused oxygen vacancy defects [19]. Moreover, the O 1s peaks were deconvoluted into two sub-peaks corresponding to the lattice oxygen (O_L_) and oxygen deficiency (O_D_), located at 530.9 and 531.3 eV [2,27] respectively, as shown in Figure 3c. As the Al doping increases, the reduction of O_D_ peak area can be evidently observed, as confirmed in Figure 3d, which shows the decrease of the relative peak intensity [O_D_/O_L_+O_D_] of O_D_ peak from 0.22 to 0.18 with increasing Al concentration. Therefore, the reductions in the proportions of the Sn^2+^ and O_D_ peaks with Al content reflect the reductions in electron density and oxygen vacancies; the channels operate efficiently at increasingly higher I_on_/I_off_ ratios and reduced SS, and are more stable in air (see also Figure 1 and Figure 2).

**Figure 4:**
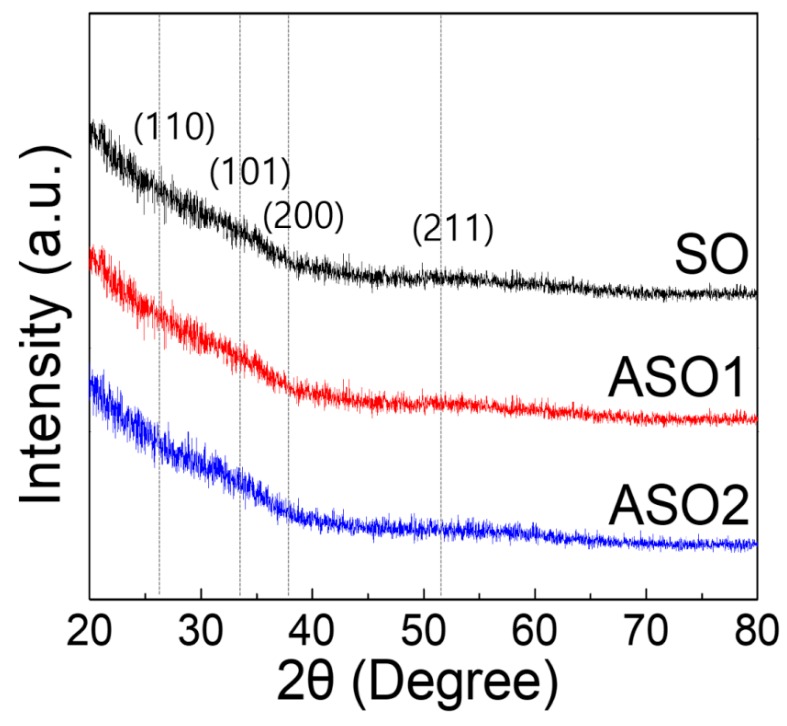
High resolution X-ray diffraction (HR-XRD) patterns of SO, SAO1, and SAO2 thin-films with different Al concentrations.

**Figure 5:**
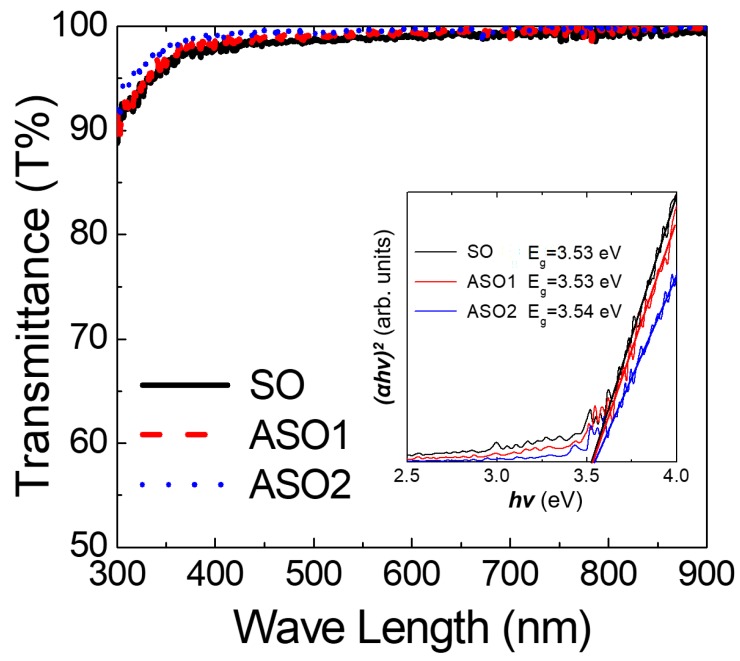
Optical transmittance values as a function of wavelength and (inset) Tauc plot as a function of photon energy (*hν*) for the SO, ASO1, ASO2 thin films with different Al concentrations.

Next, SO, ASO1, and ASO2 thin films formed on Si/SiO_2_ (100 nm) substrates were subjected to glancing-angle HR-XRD to evaluate crystallinity changes (Figure 4). The samples were prepared with the thickness of 20 nm to ensure that, if present, diffraction peaks would be evident, and annealed at 150 °C for 1 h. All samples were amorphous, lacking the diffraction peaks identified earlier [19,28]. This means our films have high areal uniformity for device characteristics, which is essential for fabricating devices on large-scale substrate [1]. To evaluate optical properties, 10 nm thick films were deposited on glass. As shown in Figure 5, all visible (λ = 390~700 nm) optical transmittances were >97%, and optical band-gaps determined by extrapolating the best fit to the intercept (at α = 0) in the Tauc plot [2,29] were ~3.5 eV plot for all samples, as required for fabrication of transparent electronic devices.

### 3.3. Adhesive Property between Al-Doped SnO_x_ Thin-Films and Plastic Substrate

**Figure 6:**
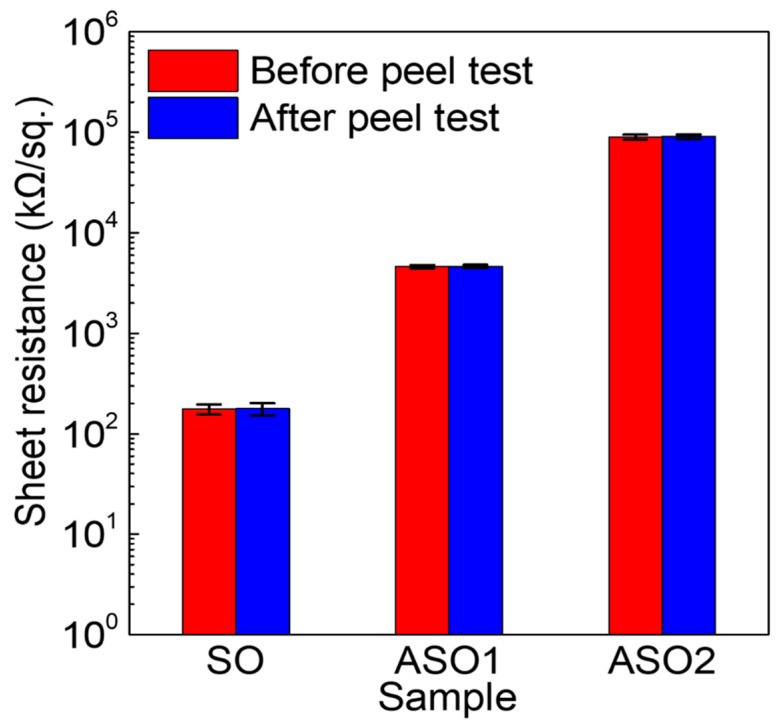
Sheet resistance changes before and after the peel test for SO, ASO1, and ASO2 thin films on a polyimide (PI) substrate.

We used the peel test to investigate the adhesion between the Al-doped SnO_x_ thin-films and PI substrates that are known to be stable below 260 °C. 10 nm thick SO, ASO1, and ASO2 thin films were RF-sputtered onto pre-cleaned PI and annealed at 150 °C. The peel test was performed using duct tape with an adhesion strength ≥ 0.7 kgf/mm (as revealed by a 180° peel test using the samples and the tape). The tape remained perfectly bonded to the SO, ASO1, and ASO2 surfaces under a vertical load of 5 kg, and was then detached. Figure 6 shows the sheet resistance values before and after the peel test. For the SO, ASO1 and ASO2 films, these were ~177, ~4613 and ~89,742 kΩ/sq. prior to the peel test, respectively. The sheet resistance of fabricated samples gradually increased with increasing Al content, attributable to the decreased carrier concentrations and improved semiconductor characteristics (see Figure 1). No significant change was evident after the peel test. If peeling occurred at the interface between a thin film and the polymer substrate, the film sheet resistance would increase. Although we did not measure the maximal adhesive strength, this was >0.7 kgf/mm, thus above the adhesive strength of duct tape.

## 4. Conclusions

We fabricated SnO_x_-based TFTs doped with different levels of Al via RF reactive co-sputtering. Compared to SnO_x_, the Al-doped films exhibited improved channel semiconductor characteristics, including a shift in V_th_ to almost 0 V, an increased I_on_/I_off_ ratio, a reduced SS, and improved stability in air, which are attributable to the reduced oxygen deficiencies (because Al is a stronger oxidizer than Sn). The oxygen deficiency reduction by Al doping was confirmed by the decrease of Sn^2+^ and O_D_ proportion in Sn 3d_5/2_ and O 1s XPS spectral analysis. The Al-doped SnO_x_ films are amorphous and have an optical transmittance of ~97% and an adhesive strength (to PI) of >0.7 kgf/mm, which facilitates their use in flexible and transparent electronics.

## Figures and Tables

**Table 1:** Electrical parameters revealed by the thin-film transistor (TFT) transfer curves scanned with forward bias sweep, hysteresis (H) in clockwise direction and Al atomic concentrations from X-ray photoemission spectroscopy (XPS) spectral analyses.

Sample	V_th_ [V]	I_on_/I_off_	μ_sat_ [cm^2^ V^−1^ s^−1^]	SS [V dec^−1^]	H [V]	Al [at %]
SO	−13.4	3.11 × 10^6^	2.24	2.37	2.67	0
ASO1	−8.0	1.38 × 10^7^	2.30	1.28	2.15	1.20
ASO2	−1.0	7.86 × 10^7^	2.24	0.68	1.13	2.24

## Supplementary Materials

The following are available online at http://www.mdpi.com/1996-1944/12/1/137/s1, Figure S1: The (I_DS_)^1/2^ versus V_G_ curves of SO, ASO1, and ASO2 TFTs for comparison of electron mobilities at V_D_ = 10.1V.
