# Supplementary material for: Electrical, Structural, Optical, and Adhesive Characteristics of Aluminum-Doped Tin Oxide Thin Films for Transparent Flexible Thin-Film Transistor Applications
Source: Materials (Basel). 2019 Jan 3;12(1):137. doi: 10.3390/ma12010137 (PMC6337128; doi:10.3390/ma12010137)
Supplement: Supplementary file 1 [file materials-12-00137-s001.pdf]

materials

MDPI

Supplementary

Electrical, Structural, Optical and Adhesive Characteristics of Aluminum-Doped Tin Oxide Thin Films for Transparent Flexible Thin-Film Transistor Applications

Seung-Hun Lee [1,4], Kihwan Kwon [1,3], Kwanoh Kim [1], Jae Sung Yoon [1,2], Doo-Sun Choi [1], Yeongeun Yoo [1,2], Chunjoong Kim [4], Shinill Kang [3,*] and Jeong Hwan Kim [1,2,*]

1. Department of Nano Manufacturing Technology, Korea Institute of Machinery and Materials (KIMM), Daejeon 34103, Republic of Korea; qwpo45ei@kimm.re.kr (S.-H.L.); nankkh@kimm.re.kr (K.K.); kkim@kimm.re.kr (K.K.); jaesyoon@kimm.re.kr (J.S.Y.); choids@kimm.re.kr (D.-S.C.); yeyoo@kimm.re.kr (Y.Y.)
2. Department of Nano-Mechatronics, University of Science and Technology, Daejeon, 34113, Republic of Korea
3. School of Mechanical Engineering, Yonsei University, Seoul, 03722, Republic of Korea
4. Department of Materials Science and Engineering, Chungnam National University, Daejeon, 34134, Republic of Korea; ckim0218@cnu.ac.kr (C.K.)

* Correspondence: jkim@kimm.re.kr (J.H.K.); snlkang@yonsei.ac.kr (S.K.)

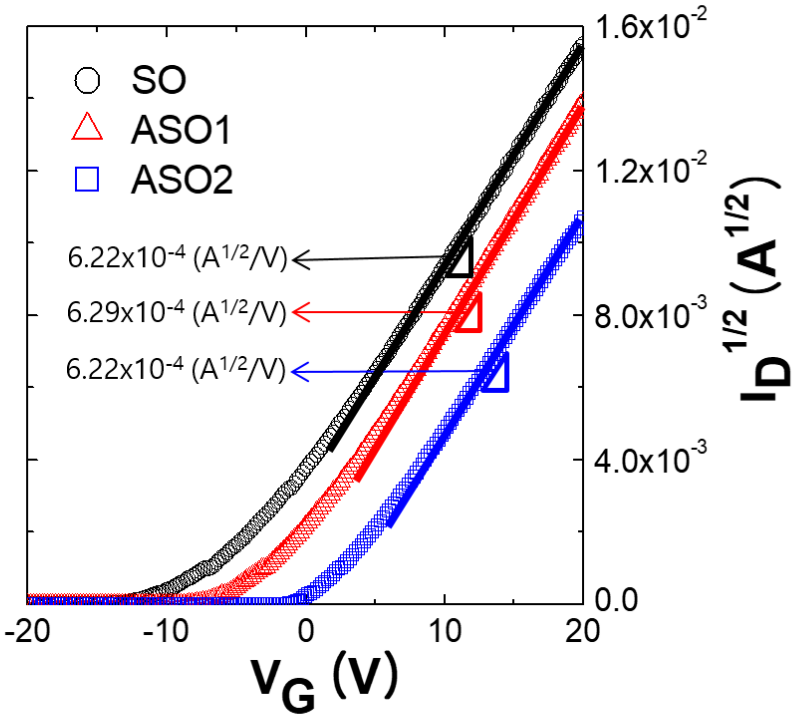

Figure S1. The $(I_{DS})^{1/2}$ versus V_G curves of SO, ASO1, and ASO2 TFTs for comparison of electron mobilities at V_D = 10.1V.
